# Effect of Blood Volume in Standard Anaerobic Blood Culture Bottles of the BacT/ALERT 3D System Used for the Detection of Pathogens and Time to Detection

**DOI:** 10.1371/journal.pone.0116728

**Published:** 2015-02-03

**Authors:** Seong Chun Kim, Sunjoo Kim, Dong-Hyun Lee, Sae-Rom Choi, Jeong-Sook Kim

**Affiliations:** 1 Department of Emergency Medicine, Institute of Health Sciences, Gyeongsang National University School of Medicine, Jinju, Republic of Korea; 2 Department of Laboratory Medicine, Institute of Health Sciences, Gyeongsang National University School of Medicine, Jinju, Republic of Korea; 3 Department of Nursing, Jinju Health College, Jinju, Republic of Korea; University Hospital San Giovanni Battista di Torino, ITALY

## Abstract

**Background:**

Blood volume may profoundly affect the isolation of microorganisms in blood cultures. The effect of blood volume in standard anaerobic bottles of the BacT/ALERT 3D system was investigated.

**Methods:**

Adult patients who visited the emergency department and referred for blood culture (n = 824) were enrolled from June to September 2013. Two sets of blood cultures were obtained from each patient. One set consisted of 5 mL that was collected in a standard aerobic bottle (SA5), 5 mL that was collected in a standard anaerobic bottle (SN5), and 10 mL that was collected in a standard anaerobic bottle (SN10). The growth of clinically significant pathogens and the time to detection (TTD) were compared between the SN5 and SN10 samples.

**Results:**

Increasing the volume of blood collected from 5 to 10 mL yielded a 14.7% improvement in the isolation of microorganisms. There was a statistically significant difference in the isolation of pathogens (14 vs. 30, *P* = 0.023) between the SN5 and SN10 samples. Gram-positive microorganisms were detected earlier in the SN10 samples than the SN5 samples (P = 0.052). The mean TTD of all pathogens was 13.5 h for the SN5 samples and 12.9 h for the SN10 samples (*P* = 0.099).

**Conclusion:**

Increased blood volume in the SN bottle yielded a significantly higher pathogen detection rate. However, there was no difference in the frequency of earlier detection or TTD between the SN5 and SN10 samples.

## Introduction

Blood culture is essential for the diagnosis of sepsis. There are several parameters that impact the success of blood culture, including blood collection time, blood sample amount, the number of blood collection sets, and skin disinfection. However, optimal blood sample amount might be the most important factor [1, 2] because bacteremia levels can be very low [3, 4, 5]. Weinstein et al [6] reported that the microorganisms isolated were significantly different when blood volume was increased in an aerobic culture bottle. We could not find a report in the literature of the effect of blood volume on microorganism isolation in an anaerobic culture bottle. Automatic blood culture machines are routinely used in large laboratory settings, and broth media are continuously developed. Therefore, an investigation of the impact of blood volume on the isolation of microorganisms is worthwhile [5]. Early reporting of blood culture results is crucial to improve morbidity and shorten the hospital stay of septic patients [2]. Time to detection (TTD) is defined as the time span from the entry of culture bottles to the observation of a positive signal in the blood culture machine. We used a BacT/ALERT 3D system (bioMererieux Inc., Durham, NC, USA) with standard aerobic (SA) and standard anaerobic (SN) bottles for blood cultures. The SA bottle has a fixed volume, and 5 mL of blood was collected in each SA bottle. Blood samples (5 mL and 10 mL) were collected in two different SN bottles to observe the effect of increased blood volume in anaerobic bottles on the isolation capability and speed of detection of microorganisms in an automatic blood culture system.

## Materials and Methods

### Study setting

This study was performed in a tertiary referral hospital. Adult patients (≧ 18 years old) who visited the emergency department and who were referred for blood cultures (n = 824) were enrolled from June to September 2013. For each venipuncture, 20 mL of peripheral blood was collected. Two sets of blood cultures were performed for each patient. One set consisted of 5 mL that was collected in a standard aerobic bottle (SA5), 5 mL that was collected in a standard anaerobic bottle (SN5), and an additional 10 mL that was collected in another standard anaerobic bottle (SN10). The actual amount of blood in each bottle was determined by dividing the weight difference between each bottle and the average of 50 blood-free bottles by blood density (1.055 g/mL).

### Blood culture procedure

We informed the medical technicians in the emergency department of the skin disinfection requirement and the amount of blood to collect before and during the study period. For each set, 20 mL of blood was recommended for collection, and 0.5% chlorhexidine alcohol was used for skin disinfection. Blood culture bottles were transferred to the clinical microbiology laboratory and incubated in a BacT/ALERT 3D automated blood culture system for 5 days. Bottles that showed a positive signal in the Bact/ALERT 3D system were routinely subjected to Gram staining and subcultured on blood agar plates, MacConkey agar plates, and chocolate agar plates. Anaerobic cultures were performed when no growth was observed in the aerobic culture, and the Gram stain was positive. Colonies were identified using the Vitek-2 system (bioMererieux Inc., Durham, NC, USA). An instrument false positive was defined as a positive signal in the BacT/ALERT 3D system with no growth of bacteria on the 3 types of agar plates. A software program automatically recorded the TTD.

### Case review

We reviewed all positive blood culture results that were obtained during the study period. An emergency department physician reviewed medical records to differentiate true bacteremia from skin contaminations in isolates. Gram-negative organisms or yeasts were always considered true pathogens. *Staphylococcus aureus*, group B *Streptococcus*, and *Enterococcus* spp. of Gram-positive cocci and *Bacillus* spp., *Corynebacterium* spp., *Micrococcus* spp., and *Propionibacterium* spp. were always considered skin contaminants regardless of the number of positive sets [2, 7]. The clinical significance of the coagulase-negative *Staphylococci* and *Streptococcus viridians* groups was carefully determined using a medical record review based on clinical signs and symptoms, laboratory findings (such as abnormal complete blood counts, C-reactive protein, and procalcitonin), radiological findings (such as pneumonic infiltrates on X-ray or CT scan), and culture results of other body specimens (such as urine, sputum, abscess, cerebrospinal fluid, and bone biopsy).

### Statistical analysis

The frequencies of clinically significant microorganisms and the frequency of earlier detection in the SN5 and SN10 samples were analyzed using a binomial test. The assumption of normality was examined using the Kolmogorov-Smirnov test. Differences in time to detection were evaluated using a Wilcoxon’s signed ranks test. All statistical analyses were performed using SPSS, version 21 (IBM Corp, Armonk, NY, USA), and differences were considered statistically significant at a *P*-value less than 0.05.

### Ethics statement

The institutional review board at the Gyeongsang National University Hospital approved this study, and informed consent was waived because blood cultures were drawn from adult patients with suspected bacteremia or fungemia upon physician request as part of routine patient care without additional tests. The clinical isolates data were de-identified and analyzed anonymously.

## Results

### Blood culture results

**Figure 1:**
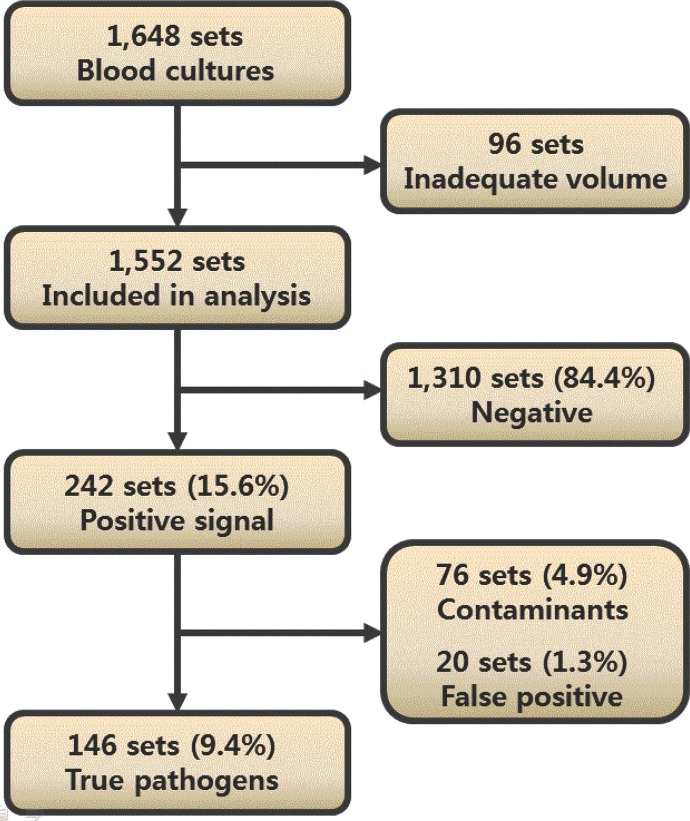
Characterization of blood culture results. False positive indicates no microorganism growth for a positive signal in the instrument.

A total of 1,648 blood culture sets were collected over 3 months. A total of 96 sets did not contain at least 80% of the required volume, and 1,552 sets were included in analyses. The average (±SD) volume was 5.66 ± 0.79 mL in the SN5 samples and 10.27 ± 0.89 mL in the SN10 samples. A positive signal was detected in 242 sets (15.6%). Skin contaminations were observed in 76 of these 242 sets (4.9%), and instrument false positives were noted in 20 sets (1.3%) (Fig. 1). Instrument false positives were more prominent in the SN10 samples (15 sets) than the SN5 (5 sets) or SA5 (4 sets) samples. Two sets showed instrument false positives in all three bottles.

### Comparison of pathogens between SN5 and SN10

Clinically significant pathogens were isolated from 146 sets (9.4%). A total of 106 of the SA5 samples were positive (6.8%), 109 of the SN5 samples were positive (7.0%), and 125 of the SN10 samples were positive (8.1%). Fourteen of the 139 isolates in the anaerobic bottles (10.1%) only grew in the SN5 samples, and 30 (21.6%) only grew in the SN10 samples (*P* = 0.023) (Table 1). There were no statistical significances, but *Escherichia coli* and Gram-positive bacteria were more frequently isolated from the SN10 samples than from the SN5 samples. A total of 95 isolates grew in both the SN5 and SN10 samples, and Gram-positive and Gram-negative bacteria accounted for 23 (24.2%) and 71 (74.7%) isolates, respectively.

**Table 1:** Clinically significant pathogens isolated from either SN5 or SN10 samples.

	Both positive	SN5 only	SN10 only	*P*
Gram-positive	23	2	9	0.065
*Staphylococcus aureus*	10	1	2	1.000
Coagulase-negative *Staphylococci*	0	0	1	NA
*Streptococcus* spp.	5	1	1	1.000
*Enterococcus* spp.	5	0	2	0.500
Gram-positive bacilli	3	0	1	NA
Other Gram-positive	0	0	2	0.500
Gram-negative	71	12	21	0.163
*Escherichia coli*	45	3	10	0.092
*Klebsiella pneumoniae*	12	5	5	1.000
*Acinetobacter baumannii*	0	3	2	1.000
Other *Enterobacteriaceae*	11	0	1	NA
Other Gram-negative	3	1	3	0.625
Fungi	1	0	0	NA
Total	95	14	30	0.023

SN5 or SN10 indicates that 5 mL or 10 mL of blood was collected in standard anaerobic bottles. Statistical comparisons were conducted using binomial tests.

### Earlier detection

Earlier growth was noted for 38 microorganisms in the SN5 samples than in the SN10 samples, and 54 microorganisms grew earlier in the SN10 samples than in the SN5 samples (*P* = 0.117) (Table 2). There was no statistically significant difference in the frequency of earlier detection between the SN5 and SN10 samples for *S. aureus, E. coli, K. pneumoniae*, or any Gram-negative microorganisms. Gram-positive microorganisms tended to be detected earlier in the SN10 samples than the SN5 samples (*P* = 0.052).

**Table 2:** Earlier detection of microorganisms grown in both standard anaerobic 5 mL (SN5) and standard anaerobic 10 mL (SN10) bottles.

	N	Earlier growth in SN5	Earlier growth in SN10	*P*
*Staphylococcus aureus*	10	2	8	0.109
*Eschericia coli*	44	19	25	0.451
*Klebsiella pneumoniae*	12	7	5	0.774
Gram-positive organisms	22	6	16	0.052
Gram-negative organisms	70	32	38	0.550
All microorganisms	92	38	54	0.117

One set that showed no difference in TTD between SN5 and SN10 and two sets with no record for TTD were not included. Statistical comparisons were conducted using binomial tests.

### Comparison of TTD between SN5 and SN10 samples

The mean TTDs for all pathogens (N = 93) were 13.5 h for the SN5 samples, and 12.9 h for the SN10 samples (*P* = 0.099, Table 3). There was no significant difference in the TTDs of Gram-positive (*P* = 0.211) or Gram-negative (*P* = 0.330) microorganisms. The most common isolates, *S. aureus, E. coli*, and *K. pneumoniae*, also showed no statistically significant difference in the TTDs between the SN5 and SN10 samples.

**Table 3:** Comparison of mean (SD) time (h) to detection of microorganisms grown in both standard anaerobic 5 mL (SN5) and standard anaerobic 10 mL (SN10) bottles.

	N	SN5	SN10	*P*
*Staphylococcus aureus*	10	15.0 (7.2)	14.7 (8.0)	0.241
*Eschericia coli*	45	11.4 (16.1)	10.7 (13.2)	0.174
*Klebsiella pneumoniae*	12	8.4 (3.9)	8.7 (4.7)	0.387
Gram-positive organism	22	14.8 (15.3)	13.3 (9.9)	0.211
Gram-negative organism	71	13.1 (18.1)	12.8 (18.2)	0.330
All microorgnism	93	13.5 (17.3)	12.9 (16.6)	0.099

Two sets with no record for TTD were not included. Statistical comparisons were conducted using Wilcoxon’s signed ranks test.

### Skin contaminants

There was no significant difference in the incidence of skin contaminants between the 3 bottles (SA5, 34 sets; SN5, 33 sets; SN10, 31 sets). Skin contaminants included 19 coagulase-negative *Staphylococci*, 17 *Bacillus* spp., 11 *Staphylococcus epidermidis*, 8 *Micrococcus* spp., 7 *Propionibacterium* spp., 3 *Corynebacterium* spp., 3 *Streptococcus gallolyticus*, 2 *Streptococcus alactolyticus*, 1 *Streptococcus dysgalactiae*, 1 *Staphylocccus caprae*, 1 *Staphylocccus constellatus*, 1 *Staphylococcus haemolyticus*, and 1 *Staphylococcus saprophyticus*. Many skin contaminants were concomitantly isolated from multiple bottles. Two *S. mitis/oralis*, two *S. dysgalactiae*, and one *S. spidermidis* were considered true pathogens by a scrutinized medical review.

## Discussion

More microorganisms were isolated in higher blood volume samples in previously published studies [6, 8]. Low-level bacteremia is common in small children [4] or unselected patients compared to neutropenic patients [3]. Kellogg et al. [4] reported that the proportion of children with a bacteremia level of ≤ 1 CFU/mL was 23%, and the proportion of children with a bacteremia level of ≤ 10 CFU/mL was 60%, which indicates a larger blood volume is necessary to detect microorganisms, even in children. The bacteremia level in the children in their study had a wider range in anaerobic bottles than aerobic bottles [4]. Our study group was adults, but the culture results may have been largely affected by the bacteremia levels in the samples in the same manner as Kellog et al. A total of 20–30 mL of blood should be taken during each venipuncture [1], but many institutions collect much less [9, 10]. A total of 45 mL was typically taken for each blood sample set in a study before the introduction of the automated blood culture systems [3]. Our study size and time period were different from Weinstein’s SA study [6], but we obtained a much higher positive rate and skin contamination rate. SA10 samples yielded significantly more *E. coli* and other bacteria of the *Enterobacteriaceae* family in the SA study, but neither Gram-positive bacteria nor non-fermentative Gram-negative rods [6] were identified. The sample size was much smaller in our SN study, but *E. coli*, Gram-positive bacteria, and total microorganisms were isolated more commonly in the SN10 samples. The increase in isolates that was attributed to increases in the collected blood volume in the SN bottles was 14.7%, which was more prominent than the 7.2% in the SA study [6]. Only 4 absolute anaerobes that were grown in the anaerobic bottles were isolated. Most of the bacteria that grew only in the SN bottles were facultative anaerobes. Therefore, we suggest that anaerobic bottles should be used to isolate facultative bacteria, rather than absolute anaerobes because these bacteria do not grow in aerobic bottles. Polymicrobial bacteremia was noted in 4 sets. Bacteria that grew only in the SN10 samples outnumbered bacteria that grew only in the SN5 samples. However, it is difficult to explain why some bacteria grew only in the SN5 samples. It is possible that bacteria were not evenly distributed in the blood or syringe, which resulted in the growth of certain bacteria only in smaller blood volume samples.

The frequency of earlier detection was more prominent in the SN10 than the SN5 samples, but the TTDs for the SN10 and SN5 samples were similar. This phenomenon was also observed in the previous SA study [6]. These authors suggested a suboptimal blood:broth ratio to dilute any antibiotics and a prolonged lag phase when culturing larger blood volume samples [6]. Regardless, we found no notable differences in TTDs for the SN bottles with blood volumes in the 5–10 mL range. We may assume that the bacteremia level of an microorganism is very high when the difference in the TTD of that microorganism is very minimal between an SN5 and an SN10 sample. Therefore, the bacteremia level of *K. pneumoniae* might be higher than *E. coli*. The greater capability of *E. coli* detection in the SN10 samples coincides with this assumption. Notably, the mean TTD of *K. pneumoniae* was even shorter in the SN5 samples. Hematological malignancy patients with neutropenia or patients with severe sepsis might have a higher bacteremia level than unselected patient groups [3, 5]. Brown and Warren [3] suggested that Gram-negative rods have a lower bacteremia level. However, this assumption does not correlate with our results because the difference in the TTD for Gram-negative rods between the SN5 and the SN10 samples was shorter than Gram-positive bacteria in this study.

The skin contamination rate was rather high (4.7%) in our study, but this rate included data from all three bottles. Notably, there was no difference in the skin contamination rate between the SN5 and SN10 samples, but instrument false positives were notably higher in the SN10 samples (15 sets) than in the SN5 samples (5 sets). A blood:broth ratio less than 1:9 might increase the instrument false positive rate. Failure to complete disinfection of the skin surface may be the main reason for the growth of skin contaminants rather than the blood volume collected. Continuous quality control of the blood culture procedure, especially the blood volume collected and skin disinfection, is mandatory [2, 10, 11].

There were several limitations to this study. The sample size was small which generated limited data that was not sufficient to draw solid conclusions. Additionally, the differentiation of true pathogens from skin contaminants was rather subjective. We only reviewed clinical data for coagulase-negative *Staphylococci* and *S. viridians*. However, the proportion of under-filled bottles was approximately 5.8%, which might have affected the results to some extent. We asked the phlebotomists to collect blood for the SN10 sample first because we experienced many failures in the collection of the desired blood volume in the preliminary study. The order of blood collection might have affected the results because the bacterial distribution in the syringe might be variable.

## Conclusions

Increasing the volume of blood collected from 5 to 10 mL yielded a 14.7% improvement in the isolation of microorganisms without affecting the skin contamination rate in the anaerobic bottles. In addition, the higher blood volume yielded significantly more pathogens. Earlier growth was more common in the SN10 than the SN5 samples, but there was no statistically significant difference in the TTD between the two groups.
